# Insect vector manipulation by a plant virus and simulation modeling of its potential impact on crop infection

**DOI:** 10.1038/s41598-022-12618-2

**Published:** 2022-05-19

**Authors:** Hyoseok Lee, Andrew P. Stephanus, Trevor M. Fowles, William M. Wintermantel, John T. Trumble, Robert L. Gilbertson, Christian Nansen

**Affiliations:** 1 Department of Entomology and Nematology, University of California, Davis, CA 95616 USA; 2 US Department of Agriculture, Agricultural Research Service, Salinas, CA 93905 USA; 3 Department of Entomology, University of California, Riverside, CA 92521 USA; 4 Department of Plant Pathology, University of California, Davis, CA 95616 USA

**Keywords:** Behavioural ecology, Ecological epidemiology, Ecological modelling

## Abstract

There is widespread evidence of plant viruses manipulating behavior of their insect vectors as a strategy to maximize infection of plants. Often, plant viruses and their insect vectors have multiple potential host plant species, and these may not overlap entirely. Moreover, insect vectors may not prefer plant species to which plant viruses are well-adapted. In such cases, can plant viruses manipulate their insect vectors to preferentially feed and oviposit on plant species, which are suitable for viral propagation but less suitable for themselves? To address this question, we conducted dual- and no-choice feeding studies (number and duration of probing events) and oviposition studies with non-viruliferous and viruliferous [carrying beet curly top virus (BCTV)] beet leafhoppers [*Circulifer tenellus* (Baker)] on three plant species: barley (*Hordeum vulgare* L.), ribwort plantain (*Plantago lanceolata* L.), and tomato (*Solanum lycopersicum* L.). Barley is not a host of BCTV, whereas ribwort plantain and tomato are susceptible to BCTV infection and develop a symptomless infection and severe curly top symptoms, respectively. Ribwort plantain plants can be used to maintain beet leafhopper colonies for multiple generations (suitable), whereas tomato plants cannot be used to maintain beet leafhopper colonies (unsuitable). Based on dual- and no-choice experiments, we demonstrated that BCTV appears to manipulate probing preference and behavior by beet leafhoppers, whereas there was no significant difference in oviposition preference. Simulation modeling predicted that BCTV infection rates would to be higher in tomato fields with barley compared with ribwort plantain as a trap crop. Simulation model results supported the hypothesis that manipulation of probing preference and behavior may increase BCTV infection in tomato fields. Results presented were based on the BCTV-beet leafhopper pathosystem, but the approach taken (combination of experimental studies with complementary simulation modeling) is widely applicable and relevant to other insect-vectored plant pathogen systems involving multiple plant species.

## Introduction

Most plant viruses require insect vectors (e.g., aphids, beetles, leafhoppers, thrips, and whiteflies) to be transmitted among plants^1^. Efficiency and dynamics of plant virus acquisition and transmission by insect vectors are likely shaped by plant virus-insect vector coevolution^2,3^. Insect vectors of plant viruses exhibit innate host preference to optimize fitness and that of their offspring^4,5^, but such fitness optimization may not always align with host adaptation of the plant viruses they transmit^6,7^. Consequently, insect vector manipulation should be viewed as a complex of conflicting selection pressures and optimizations between insect vectors and the plant viruses they transmit. Accordingly, plant viruses have evolved strategies to manipulate their insect vectors to prefer plant species suitable for virus propagation. Several important reviews have described these manipulations of both host plants and insect vectors by plant pathogenic microorganisms^8–10^. Throughout this article, “insect vector manipulation”, as opposed to “innate host plant preference”, refers to plant viruses manipulating feeding and oviposition of their insect vector in ways that are perceived to enhance fitness of a plant virus.

Manipulation of feeding behavior may be expressed in terms of altered probing preference when choices of plants are available to insect vectors. Additionally, under both choice- and no-choice conditions, duration of probing events may also be altered. Plant viruses may influence both numbers and duration of leaf probing events by piercing-sucking insect vectors^11^. Plant viruses benefit from patterns of feeding behavior of insect vectors based on their modes of transmission^9^. Non- or semi-persistently transmitted plant viruses are transmitted during brief probing events, whereas persistently transmitted plant viruses require longer-lasting feeding events^12^. Thus, insect vector manipulation resulting in short and numerous probing events may optimize transmission of non- or semi-persistently transmitted plant viruses. In contrast, persistently transmitted plant viruses would have enhanced transmission by manipulating insect vectors to have longer feeding periods^8,9^. Stafford et al.^13^ showed that tomato spotted wilt virus (TSWV) manipulated probing behavior of its vector, Western flower thrips [*Frankliniella occidentalis* (Pergande)], resulting in increased frequency of leaf probing events by males and enhanced transmission of the non-phloem-limited TSWV. Additionally, it has been demonstrated that non-viruliferous green peach aphids [*Myzus persicae* (Sulzer)] prefer host plants infected with potato leaf roll virus (PLRV), whereas viruliferous conspecifics prefer non-infected plants^14^. Thus, PLRV appears to be manipulating its insect vector to maximize transmission to non-infected plants.

Regarding manipulation of oviposition, it has been demonstrated that *Bemisia tabaci* (Gennadius) Mediterranean (MED) vectoring tomato yellow leaf curl virus (TYLCV) laid more eggs and gained more weight than conspecifics feeding on non-infected plants^15^. In addition, Chen et al.^16^ found that MED preferentially settled and oviposited on TYLCV-infected plants than on non-infected plants. From an evolutionary standpoint, TYLCV benefits from manipulation of both settling and oviposition preferences, because TYLCV can also be transmitted transovarially by MED^17^. In short, a growing body of knowledge about plant virus manipulation of both plants and insect vectors provides critical insights into complex species interactions and behavioral patterns.

Once insect vector manipulation has been identified and characterized, simulation models can be developed to predict broader epidemiological implications and ultimately how such insect vector manipulation may affect crop production. A few but important studies have described use of simulation models to quantify possible effects of insect vector manipulation. For instance, Ogada et al.^18^ predicted that insect vector manipulation of Western flower thrips increased TSWV transmission rate up to 33%. In addition, simulation model can be used to characterize effects of insect vector manipulation under various environmental conditions^19^. For example, simulation model may be used to examine effects of insect vector manipulation on infection of crop plants in trap cropping systems^20^. Here, trap cropping refers to managed cropping systems, in which a given plant species is used as a “decoy” (trap crop) to attract insect pests, so that a main crop experiences reduced insect pest infestation. In some cases, trap crops can be the same as the main crop but be more attractive due to different planting date or altered management (i.e. different fertilization/irrigation regimes). A trap crop may also be a different variety or plant species with higher relative attractiveness than the main crop. In all trap cropping systems, a common factor is that insect pests are “offered choices” of feeding and oviposition plants at a landscape level. Thus, performance of a given trap cropping approach to insect pest management depends on an expected host preference of target insect pests, but insect vector manipulation may alter such innate host preference by insect-vectored pathogens.

In the present study, a phloem-limited plant virus [beet curly top virus (BCTV)] and its insect vector, the beet leafhopper [*Circulifer tenellus* (Baker) (Hemiptera: Cicadellidae)], were used as a model pathosystem to study insect vector manipulation. The beet leafhopper is the only known vector of BCTV in North America, and BCTV is transmitted in a persistent circulative manner. BCTV is the type species of the genus *Curtovirus*, family *Geminiviridae*. The beet leafhopper and BCTV have wide host ranges^21,22^, and BCTV causes yield losses in economically important crops, including tomato, sugar beet, pepper, spinach, and common bean^23,24^. The study objectives were: (1) to experimentally determine if beet leafhoppers carrying BCTV (viruliferous) show manipulated probing/oviposition preference or probing behavior on three plant species: barley (*Hordeum vulgare* L.), ribwort plantain (*Plantago lanceolata* L.), and tomato (*Solanum lycopersicum* L.), as compared with non-viruliferous beet leafhoppers. Barley is not a host of BCTV, whereas ribwort plantain and tomato are susceptible to BCTV infection and develop a symptomless infection and severe curly top symptoms, respectively. Ribwort plantain plants can be used to maintain beet leafhopper colonies for multiple generations (suitable), whereas tomato plants cannot be used to maintain beet leafhopper colonies (unsuitable)^25^ (Fig. S1); and (2) utilize a simulation model to assess potential effects of insect vector manipulation on BCTV spread in tomato fields with various percentages of barley or ribwort plantain as trap crop. We predicted insect vector manipulation effects by incorporating probing preference and behavior of viruliferous beet leafhoppers and those of non-viruliferous conspecifics as manipulated and innate preference and behavior.

Most studies of insect vector manipulation focus on a single plant species, although plant communities in natural and agricultural environments possess varying degrees of diversity. Thus, under real-world conditions, insect vectors face choices regarding feeding and oviposition plants. Furthermore, most plant viruses and their insect vectors have wide host ranges. We therefore investigated the role of insect vector manipulation in the BCTV etiology in tomato, a plant that is not a preferred plant species of the beet leafhopper but is highly suitable for BCTV.

## Materials and methods

### Insects and plants

Non-viruliferous and viruliferous beet leafhoppers were originally obtained from colonies maintained in the laboratory of R. L. Gilbertson in the Department of Plant Pathology at the University of California, Davis. The colonies were maintained in BugDorm mesh cages (61 cm × 61 cm × 61 cm, Megaview Science, Taichung, Taiwan) with non-infected sugar beet (*Beta vulgaris* L. var. saccharifera) or sugar beet infected with an isolate of the BCTV severe-type strain in separate greenhouses (25 ± 5 °C and 80 ± 10% relative humidity). BCTV-infection of sugar beet plants and beet leafhoppers was confirmed with a well-established PCR-based method^26^.

Barley (cultivar Champion), ribwort plantain, and tomato (cultivar APT 410) were grown in four-inch plastic pots with a mixture of 1:1:1:1 ratio of pumice: sand: sphagnum peat moss: redwood sawdust in a greenhouse at 25 ± 5 °C and 80 ± 10% relative humidity. All plants were watered daily and fertilized with 0.5% soluble N-P-K fertilizer (6:1:4) in 200 ml of water. One- to two-month-old plants were used for all experiments. Barley and ribwort plantain were selected among trap crop candidates based on probing preference of non-viruliferous beet leafhoppers (Fig. S2). All protocols using insects and plants in this study complied with relevant institutional, national, and international guidelines and legislations.

### Dual-choice experiments

**Figure 1:**
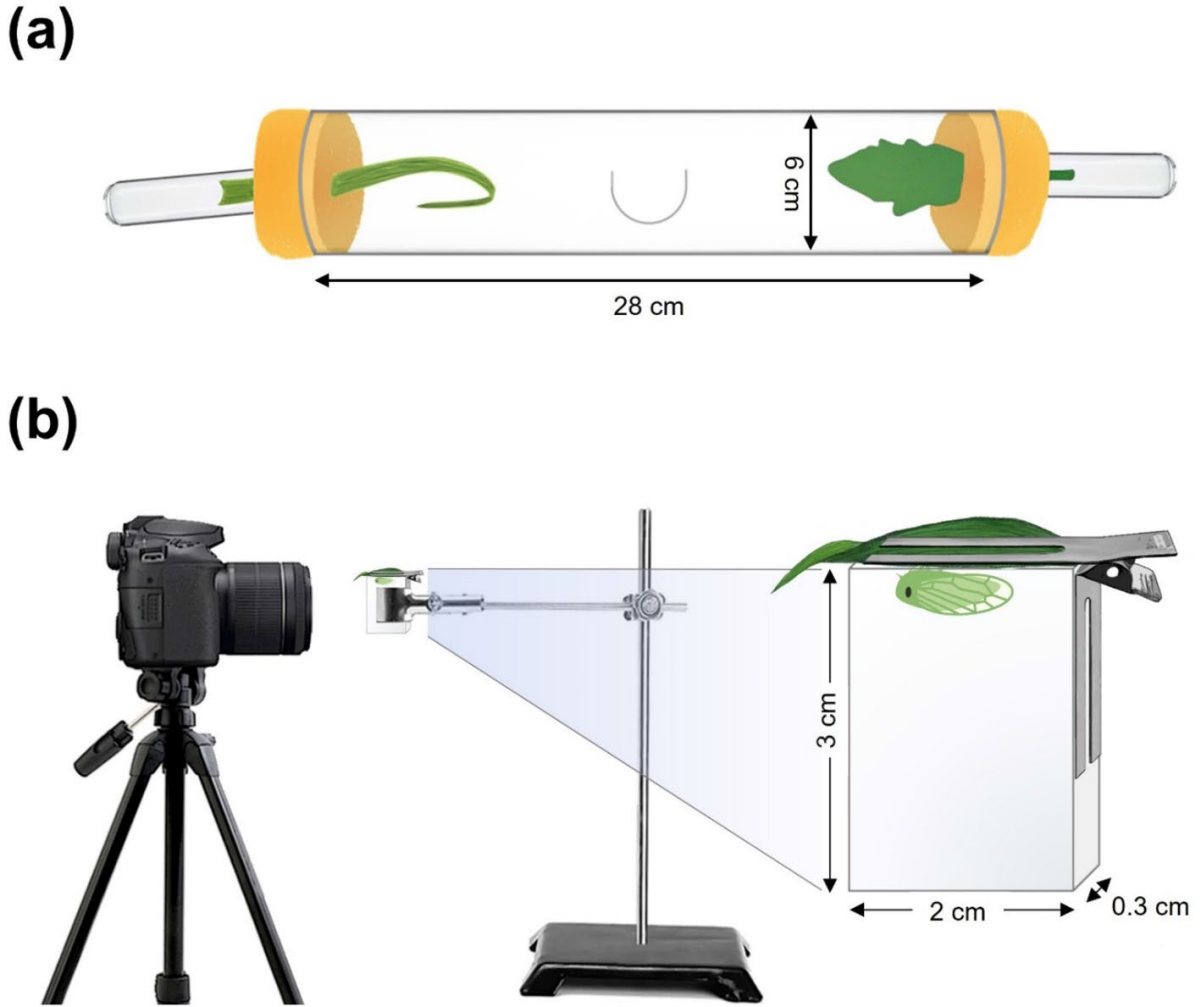
Diagrammatic illustrations of (**a**) feeding and oviposition preference experiments and (**b**) feeding behavior.

Using dual-choice experiments (i.e., tomato vs. ribwort plantain and tomato vs. barley), we investigated probing and oviposition preference of non-viruliferous and viruliferous beet leafhoppers. Dual-choice experiments were conducted in clear plastic tubes (28 cm × 6 cm; L × D), in which single leaves/leaflets were inserted in either end, and beet leafhopper individuals were released in the middle (Fig. 1a). Each leaf/leaflet was placed into a vial (8 cm × 2.5 cm; L × D) filled with water and held in place with cotton. For each dual-choice bioassay, three pairs (six individuals) of newly emerged (< 72 h) non-viruliferous or viruliferous beet leafhoppers were starved for four hours at 25 °C and then released into plastic tubes and kept in a controlled environment chamber (25 ± 0.5 °C and 50 ± 5% relative humidity). Similar to previously published studies^27,28^, probing events were counted based on McBryde staining^29^. Moreover, after 24 h of exposure to feeding beet leafhoppers, leaves/leaflets were collected and stained with McBryde’s solution^30^, which is 0.2% (wt/vol) acid fuchsin in a mixture of 95% ethanol and glacial acetic acid (1:1 vol/vol), for 24 h. Leaves/leaflets were then transferred to a clearing solution of distilled water, 99% glycerol, and 95% lactic acid (1:1:1 vol/vol/vol) at 95 °C for four hours. Stained probing events and eggs were counted under a binocular stereomicroscope (Olympus SZ51; Olympus, Tokyo, Japan).

### No-choice experiments

Total number of probing events and mean duration of each probing event by non-viruliferous and viruliferous beet leafhoppers were recorded on each plant species. Single non-viruliferous and viruliferous beet leafhoppers were starved for four hours at 25 °C and afterwards transferred to a glass cage (3 cm × 2 cm × 0.3 cm) attached to abaxial sides of leaf/leaflet from each plant species (Fig. 1b). Probing behavior was video recorded for 30 min with a Canon EOS 70D fitted with a Canon MP-E 65 mm macro lens (Canon, Huntington, NY, USA) (https://youtu.be/HLdBd4grQ34). The confined width of the cage was allowed for focusing of the camera on the mouth part of beet leafhopper. Duration of probing events was determined as the time period in which stylets were inserted into leaves/leaflets^11^.

### Statistical analyses

All statistical analyses were performed with R software (version 3.6.1)^31^. Arcsine-transformed percentages of probing events and eggs laid on each plant species were compared to determine preference of viruliferous and non-viruliferous beet leafhoppers. Insect vector manipulation was determined by using two-way analysis of variance (ANOVA) followed by Tukey’s HSD test. Significant differences were determined at the 0.05 level. Normality of data was checked with Shapiro–Wilk normality test.

Regarding data from video recordings of probing behavior, total numbers of probing events were square root transformed for statistical analysis. Normality of mean duration of probing events and transformed numbers of probing events on different plant species were examined based on Shapiro–Wilk normality test. Statistical comparison of average probing behavior by viruliferous and non-viruliferous beet leafhoppers among all plant species was based on two-way ANOVA followed by Tukey’s HSD test.

### Simulation of BCTV spread in tomato fields

Although beet leafhoppers can transmit BCTV to tomato plants, few eggs are laid on tomato plants^25^, and newly hatched nymphs don’t carry BCTV. Therefore, we only considered probing preference and duration of probing of beet leafhoppers in model simulations. We examined two tomato field scenarios with either barley or ribwort plantain as a trap crop. In both scenarios, total number of plants was held constant at 100, but percentage of trap crop plants represented percentages ranging from 0% (i.e., tomato only) to 90% (only 10% tomato plants). We assumed settlement of viruliferous beet leafhoppers to be driven by two independent variables: (1) probing preference for plant species and (2) percentages of available plant species^19^ such that:

1 $$\alpha_{y,s} = \frac{{\gamma_{s} P_{y,s} D_{s} }}{{\mathop \sum \nolimits_{s = 1}^{S} \mathop \sum \nolimits_{z = 1}^{Z} \gamma_{s} D_{s} P_{z,s} }}$$

where *α*_*y,s*_ denotes the proportion of viruliferous beet leafhoppers that probe on plant species, *s* (barley [*b*], ribwort plantain [*p*], and tomato [*t*]) of infection status *y* (non-infected [n] or infected [i]). *γ*_*s*_ denotes probing preference of viruliferous beet leafhoppers for plant species *s*. *P*_*y,s*_ denotes total number of plant species, *s,* of infection status, *y*, and *s* = 1, …, *S* indexes all plant species, while *z* = 1, …, *Z* indexes all plant infection statuses (infected or non-infected). *D*_*s*_ denotes probing duration (day) on plant species, *s*. Incorporating probing preference and probing behavior yields^19^:

2 $$\frac{{dP_{n,s} }}{dt} = - \beta \alpha_{n,s} V\quad \left( {{\text{for}}\,{\text{non-infected}}\,{\text{plant}}\,s} \right)$$

3 $$\frac{{dP_{i,s} }}{dt} = \beta \alpha_{n,s} V\quad \left( {{\text{for}}\,{\text{infected}}\,{\text{plant}}\,s} \right)$$

*β* denotes transmission rate coefficient from a viruliferous beet leafhopper to a non-infected plant and determined as *β* = 0.38. *β* value was identified as the value Stafford et al. ^32^ measured inoculation success rate of one individual beet leafhopper. *α*_*n,s*_ denotes proportion of viruliferous beet leafhoppers that probe on non-infected plant species, *s. V* denotes number of viruliferous beet leafhoppers.

Each simulation began with non-infected plants and 10 viruliferous beet leafhoppers. We quantified BCTV spread in tomato fields based on assumption of innate probing preference and behavior or presence of insect vector manipulation. Innate probing preference and behavior equals probing preference: *γ*_*i,p*_ = 0.63, *γ*_*i,t*_ = 0.37; *γ*_*i,b*_ = 0.6, *γ*_*i,t*_ = 0.4 and probing behavior: *D*_*p*_ = 0.0122, *D*_*t*_ = 0.0120, *D*_*b*_ = 0.0109. Insect vector manipulation equals probing preference: *γ*_*m,p*_ = 0.53, *γ*_*m,t*_ = 0.47; *γ*_*m,b*_ = 0.47, *γ*_*m,t*_ = 0.53 and probing behavior: *D*_*p*_ = 0.0139, *D*_*t*_ = 0.0150, *D*_*b*_ = 0.0055. Percentages of BCTV-infected tomato plants and time to 20% infection were calculated in the scenarios. All simulations were performed in R.

## Results

### Probing and oviposition preference

**Figure 2:**
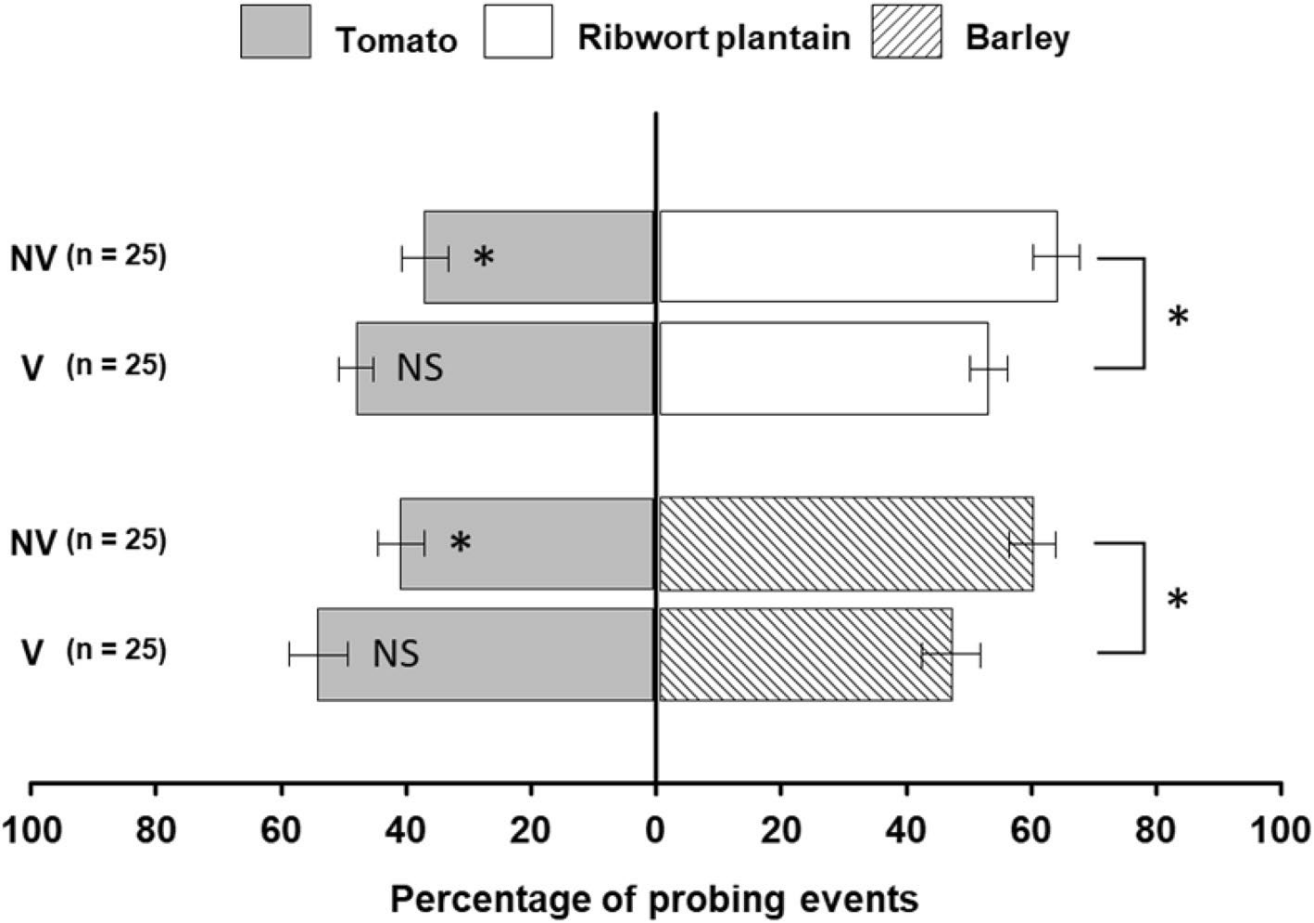
Probing events by non-viruliferous and viruliferous beet leafhoppers to leaves/leaflets in the dual-choice arena. Data are presented as mean percentages ± SE. Asterisks indicate significant differences (*p < 0.05). NS: No significant difference (*p* > 0.05).

In dual-choice experiments, percentage of leaf/leaflet probing events on each plant species was used as an indicator of probing preference by non-viruliferous and viruliferous beet leafhoppers (Fig. 2). Actual numbers of probing events and eggs laid are presented in Table S1. A significant difference in probing preference between non-viruliferous and viruliferous beet leafhoppers (*F* = 8.22, df = 1, 96, *P* = 0.005) was observed in the ribwort plantain vs. tomato combination. Thus, a post hoc Tukey’s HSD test revealed that non-viruliferous beet leafhoppers probed ribwort plantain significantly more than tomato, whereas viruliferous beet leafhoppers showed no significance (non-viruliferous: *P* < 0.001; viruliferous: *P* = 0.776). There was also a significant difference in probing preference between non-viruliferous and viruliferous beet leafhoppers in the barley vs. tomato combination (*F* = 23.98, df = 1, 96, *P* < 0.001). Here, non-viruliferous beet leafhoppers probed barley significantly more than tomato (Tukey’s HSD; *P* < 0.001), whereas viruliferous beet leafhoppers showed no preference (Tukey’s HSD; *P* = 0.298). Therefore, viruliferous beet leafhoppers were less selective in their host plant choice (i.e., reduced preference) compared to non-viruliferous conspecifics.

**Figure 3:**
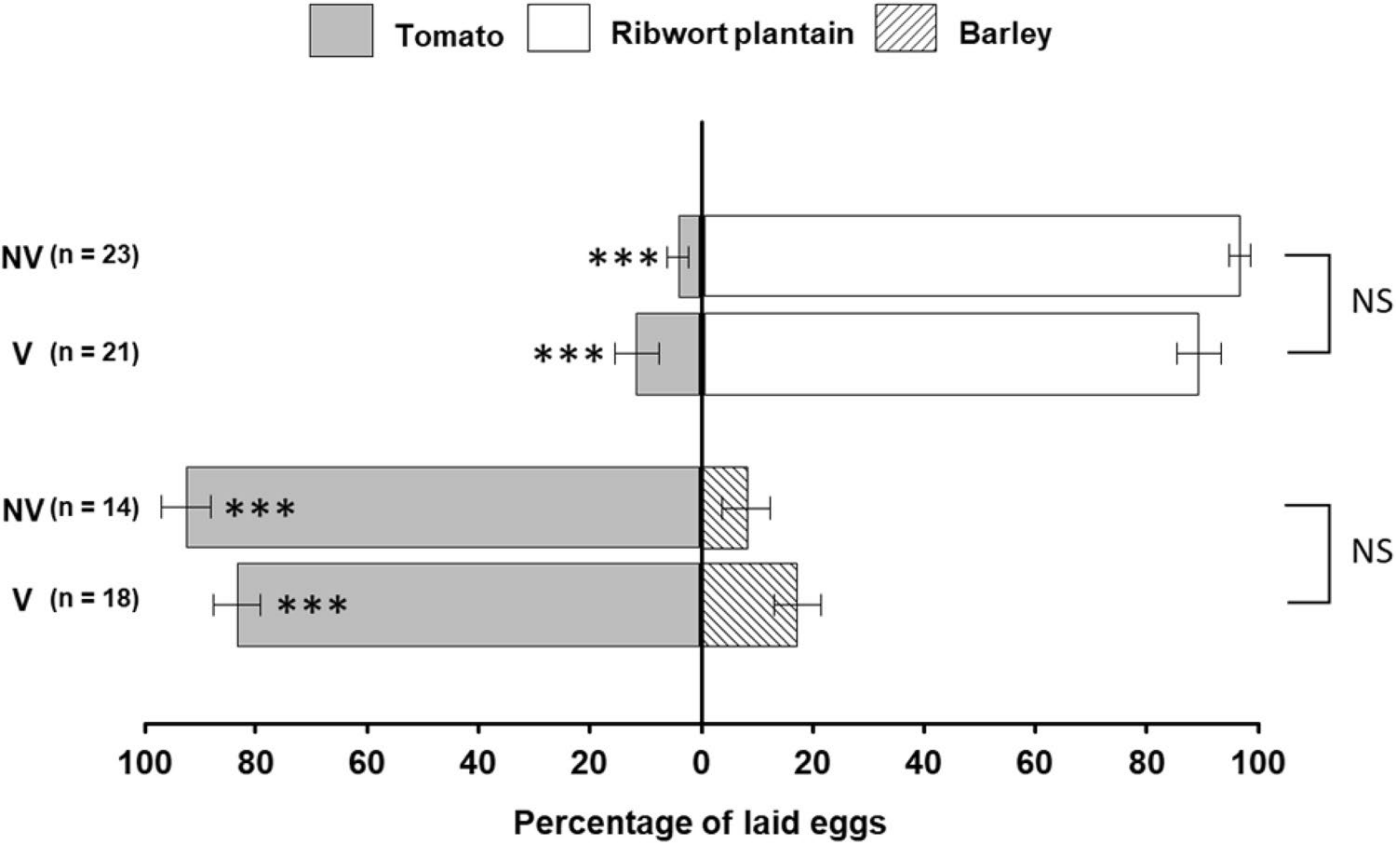
Oviposition by non-viruliferous and viruliferous beet leafhoppers on leaves/leaflets in the dual-choice experiments. Data are presented as mean percentages ± SE. Asterisks indicate significant differences (***p < 0.001). NS: No significant difference (*p* > 0.05).

Oviposition preference of non-viruliferous and viruliferous beet leafhoppers was determined by comparing percentages of eggs laid on choices between two plant species (Fig. 3). There was no significant difference in oviposition preference between non-viruliferous and viruliferous beet leafhoppers in neither the ribwort plantain vs. tomato combination (*F* = 2.76, df = 1, 84, *P* = 0.1) nor barley vs. tomato (*F* = 3.858, df = 1, 60, *P* = 0.054). A post hoc Tukey’s HSD test revealed that both viruliferous and non-viruliferous beet leafhoppers preferred ribwort plantain over tomato for oviposition (both viruliferous and non-viruliferous: *P* < 0.001). In contrast, tomato was preferred over barley by viruliferous and non-viruliferous beet leafhoppers in the tomato vs. barley combination (Tukey’s HSD; *P* < 0.001). Thus, viruliferous and non-viruliferous beet leafhoppers showed the same oviposition preference in the two combinations of plant species.

### Probing behavior on each plant species

**Figure 4:**
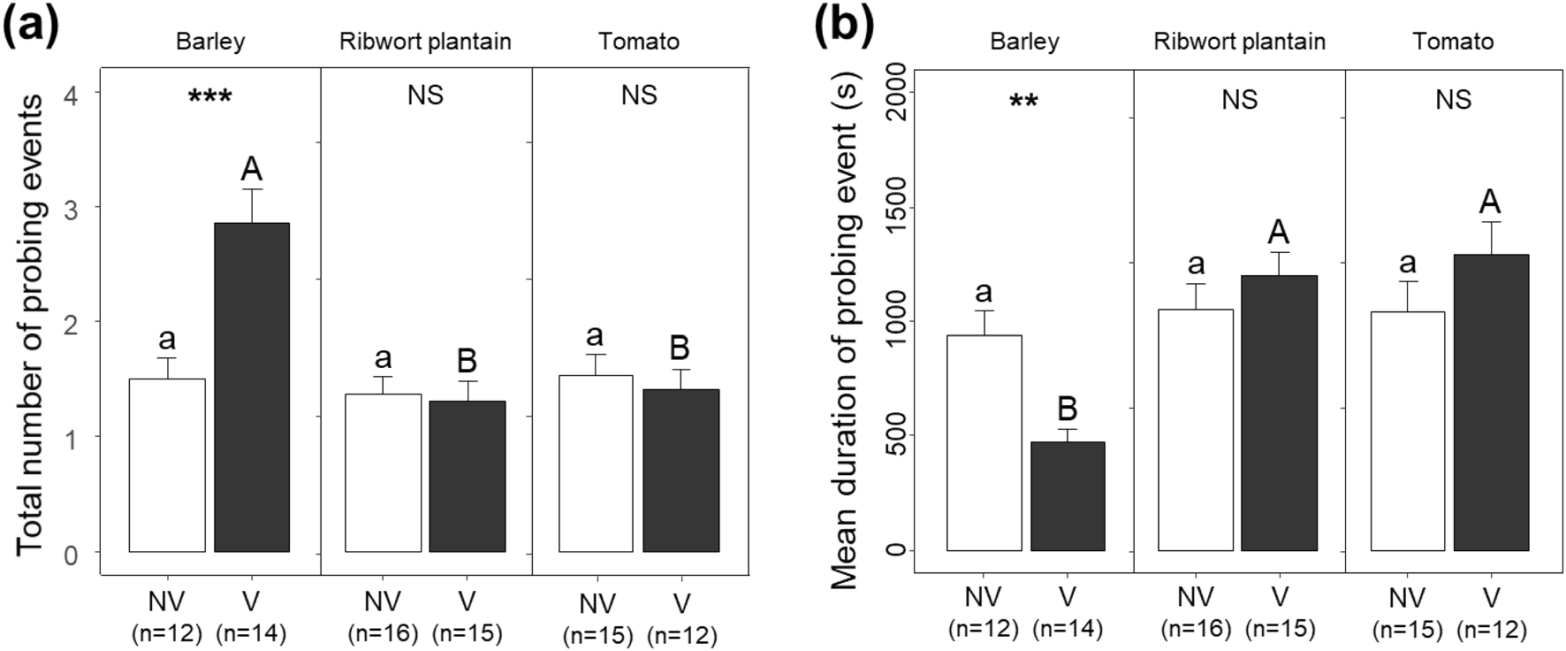
Probing events by non-viruliferous and viruliferous beet leafhoppers on barley, ribwort plantain, and tomato. (**a**) Total number of probing events; (**b**) mean duration per probing event. Letters indicate significant differences among plant species (Tukey test, *p* < 0.05). Asterisks indicate statistically significant differences between non-viruliferous and viruliferous beet leafhoppers (**p < 0.01, ***p < 0.001). NS: No significant difference (*p* > 0.05).

Viruliferous beet leafhoppers showed a significant difference in total numbers of probing events on barley compared with ribwort plantain and tomato (*F* = 7.436, df = 2, 79, *P* = 0.001) (Fig. 4a), whereas this was not seen for non-viruliferous beet leafhoppers. A post hoc Tukey’s HSD test revealed that viruliferous leafhoppers probed barley 1.9 more times than non-viruliferous conspecifics (*P* < 0.001). Moreover, only viruliferous beet leafhoppers showed a significantly reduced mean duration of probing event on barley compared with ribwort plantain and tomato (*F* = 5.122, df = 2, 79, *P* = 0.008) (Fig. 4b). The probing duration of viruliferous beet leafhoppers on barley was 0.5 times shorter than that of non-viruliferous conspecifics (Tukey’s HSD; *P* = 0.007). The difference was not observed for ribwort plantain or tomato.

### Simulation of BCTV spread in tomato fields

**Figure 5:**
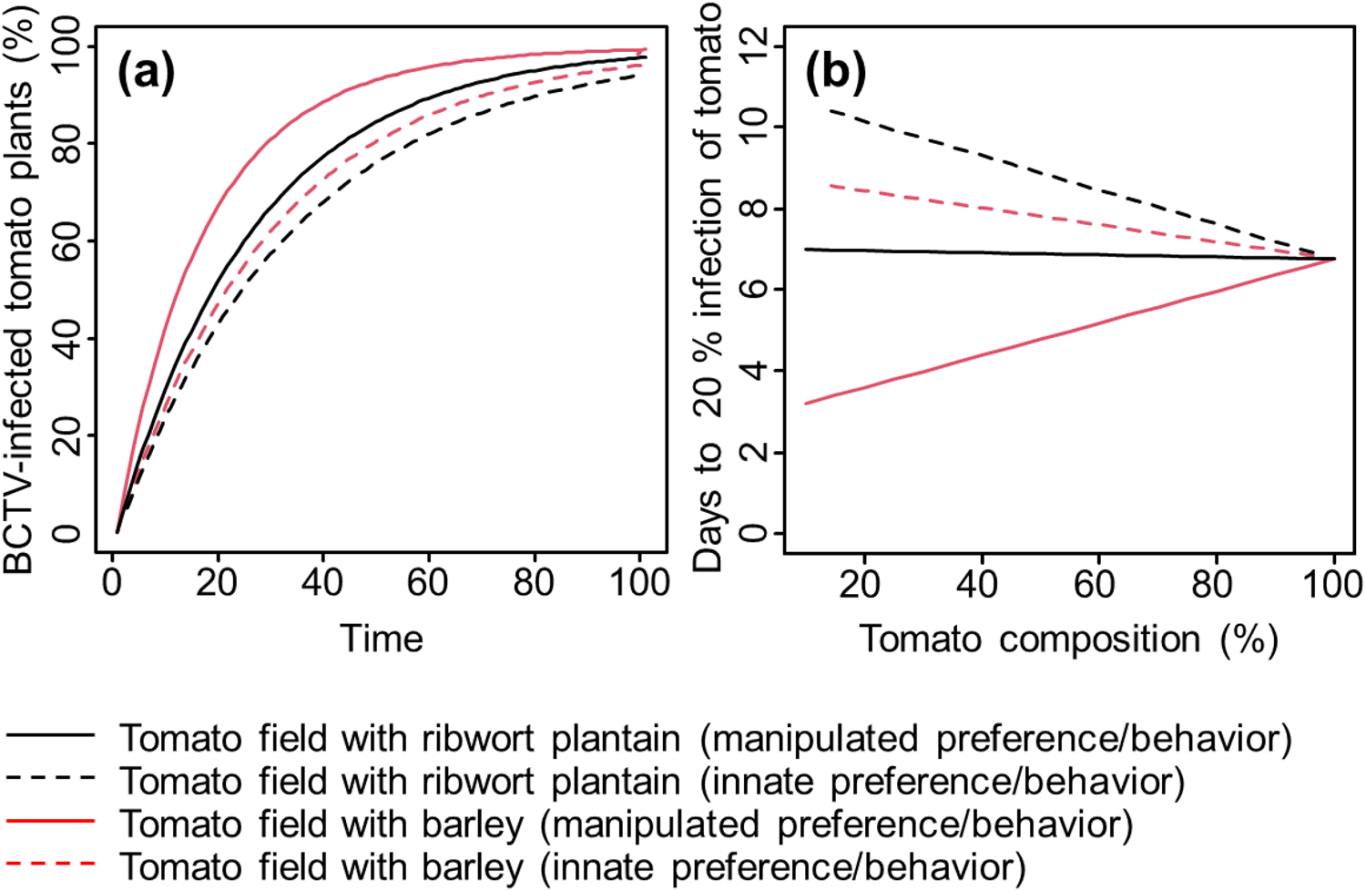
BCTV manipulation effects on (**a**) the percentage of BCTV-infected tomato plants and (**b**) the time to 20% infection of tomato plants across various tomato compositions were examined in tomato fields with ribwort plantain or barley as a trap crop. The cases with manipulated preference and innate (without manipulated) preference are shown in solid and dashed lines, respectively. Black lines represent tomato fields with ribwort plantain as a trap crop and red lines represent tomato fields with barley as a trap crop.

Based on simulation modeling of BCTV spread in scenarios with different percentages of trap crops, we observed that assumption of insect vector manipulation increased the risk of tomato plant infection in terms of the percentage of infected tomato plants and time needed to reach 20% infection (Fig. 5). Viruliferous beet leafhoppers with manipulated probing preference resulted in higher percentages of infected tomato plants than conspecifics with innate probing preference in the tomato fields with barley or ribwort plantain as a trap crop (Fig. 5a). In addition, shorter probing times on barley were associated with increased BCTV infection in tomato as compared to tomato with ribwort plantain trap crop. Insect vector manipulation was also predicted to accelerate the spread of BCTV, with the effect decreasing as the ratio of tomato plants to trap crops increased (Fig. 5b). We observed a linear relationship between trap crop percentage and BCTV spread, in which it would take 2–8 days for 20% of tomato plants to be infected depending on trap crop species. In tomato fields with barley trap crops, the time to reach 20% infection was greater than in simulations with ribwort plantain as a trap crop.

## Discussion

In this study, we demonstrated that BCTV appears to manipulate probing preference and behavior by beet leafhoppers, whereas there was no significant difference in oviposition preference. BCTV is only transmitted during probing but not transovarially transmitted from infected female beet leafhoppers to their progeny^26^. Therefore, BCTV would have less evolutionary benefit from manipulating oviposition preference versus probing preference of beet leafhoppers. Indeed, we observed that non-viruliferous beet leafhoppers preferred to probe on barley and ribwort plantain compared with tomato, whereas viruliferous beet leafhoppers showed no preference. In addition, mean probing duration was shorter for viruliferous beet leafhoppers on barley compared with non-viruliferous conspecifics. This alteration of probing preference and behavior in viruliferous beet leafhoppers may result in enhanced BCTV transmission in tomato fields, and that was examined based on a simulation model of tomato fields under different trap crop scenarios. Simulation modeling based on limited parameters predicted that BCTV infection rates would to be higher in tomato fields with barley compared with ribwort plantain as a trap crop. Simulation results supported the hypothesis that manipulation of probing preference and behavior could increase BCTV infection in tomato fields. Finally, these results suggest that beet leafhopper manipulation by BCTV following virus acquisition under natural conditions (i.e., high plant diversity) may accelerate the spread of BCTV in tomato fields. BCTV outbreaks in tomato are associated with high densities of viruliferous beet leafhoppers^33,34^. Therefore, viruliferous beet leafhopper populations, which have acquired BCTV from symptomless weeds outside tomato fields, would already have altered probing preference and behavior, further contributing to BCTV outbreaks in tomato crops.

Phytophagous insects commonly use chemical cues to locate and accept host plants^35^, which suggests that viruliferous status of insect vectors may affect how olfactory and/or gustatory cues are processed^36^. Processing of volatile plant cues by insects is mediated by soluble binding proteins, found in olfactory and gustatory organs^37^. Chemosensory proteins (CSPs) and odorant-binding proteins (OBPs) were identified as the major soluble proteins found in sensillar lymph of insects^38^. Those proteins are conserved across insect species, suggesting they may play an important role in host selection by insects. Indeed, it has been shown that these proteins affect host preference of some insects, including *Drosophila sechellia*^39^, *Adelphocoris fasciaticollis*^40^, and *Nilaparvata lugens*^41^. Thus, plant viruses may alter the expression of CSPs and OBPs, and consequently the host preference. Hu et al.^42^ reported that viruliferous *Sogatella furcifera* (carrying southern rice black-streaked dwarf virus) exhibited decreased expression levels of OBPs, which altered host preference of the viruliferous *S. furcifera*. Hence, future research comparing the expression levels of CSPs and OBPs between viruliferous and nonviruliferous beet leafhoppers may reveal molecular mechanisms of probing preference manipulation.

Viruliferous beet leafhoppers showed an increase in the number of probing events, but a decrease in the mean duration of each probing event only on barley, which is not a host for BCTV. This suggests the possibility of plant species-specific manipulation of probing behavior of viruliferous beet leafhoppers. Broadly, insects tend to shorten probing duration on less suitable plant species, which implies that mean duration of probing events can be used as an indicator of feeding host suitability^43,44^. Stafford and Walker^45^ characterized the feeding behavior of beet leafhoppers using an electrical penetration graph (EPG). They classified feeding into three phases: pathway phase, non-phloem ingestion phase, and phloem phase, and measured mean duration of each phase. In addition, time from onset of probing to phloem phase was measured for critical stylet penetration behavior associated with inoculation of BCTV^32^. Mean duration of probing events of the viruliferous beet leafhoppers on barley was similar to the median time to phloem salivation, as determined by Stafford et al.^32^. Thus, we suspect that the viruliferous beet leafhoppers rejected barley at the phloem phase, and that the chemical composition of the phloem sap may be involved in the BCTV-induced host rejection process. In future work, it would be informative to confirm in which feeding phase beet leafhoppers reject plants by using EPG.

Most insect vector manipulation studies with plant viruses include only a single host plant species with different infection statuses^46^. However, more studies are needed that incorporate multiple plant species because insect vector manipulation could be species-specific and most plant viruses have variable host ranges^47^. Shoemaker et al.^19^ found a decrease in overall plant pathogen spread through multi-host plant species due to insect vector manipulation. However, when only infection of the main crop (i.e., tomato) was considered, our simulation modeling showed an increase in the spread of BCTV. In addition, the rate of BCTV spread was affected depending on trap crop species. Therefore, multiple plant systems provide an important opportunity to better understand insect vector manipulation effects on the spread of plant viruses in natural and agricultural environments.

In summary, we experimentally examined insect vector manipulation and predicted its impact in tomato fields using simulation modeling. Although three plant species were used in this preliminary study, future work with additional plant species will be necessary to address the wide host ranges of beet leafhopper and BCTV^21,22^ and their influence on infection of tomato crops. Results presented in the current study were based on the BCTV-beet leafhopper pathosystem, and this study combining experimental studies with complementary simulation modeling can contribute to more thorough understanding of the dynamics driving not only beet leafhopper transmission of BCTV, by providing information on specific key factors contributing to virus epidemiology. Further, such a system can also be relevant to other insect-vectored plant pathogen systems involving multiple plant species.

## Supplementary Information

Supplementary Information.

## Data Availability

All data relevant to the study are included in the article or uploaded as supplementary information. Model code is available on GitHub (https://github.com/hyoseoklee23/Vector_manipulation).
