# Supplementary material for: Insect vector manipulation by a plant virus and simulation modeling of its potential impact on crop infection
Source: Sci Rep. 2022 May 19;12:8429. doi: 10.1038/s41598-022-12618-2 (PMC9119975; doi:10.1038/s41598-022-12618-2)
Supplement: Supplementary file 1 — Supplementary Information. [file 41598_2022_12618_MOESM1_ESM.docx]

**Supplementary materials**

**Figure S1.** Adult survivorship of non-viruliferous beet leafhoppers confined on barley, ribwort plantain, and tomato plants for 40 days. Newly emerged adults were reared at 27 °C, 50% relative humidity, and a photoperiod of 16:8 (L:D) h. The experiment was replicated 10 times for each plant species and 15 beet leafhoppers were used for each replication. The median longevity on barley, ribwort plantain, and tomato plants were 8.2, 42, and 2.2 days, respectively. The data for sugar beet was acquired from Munyaneza and Upton ^1^


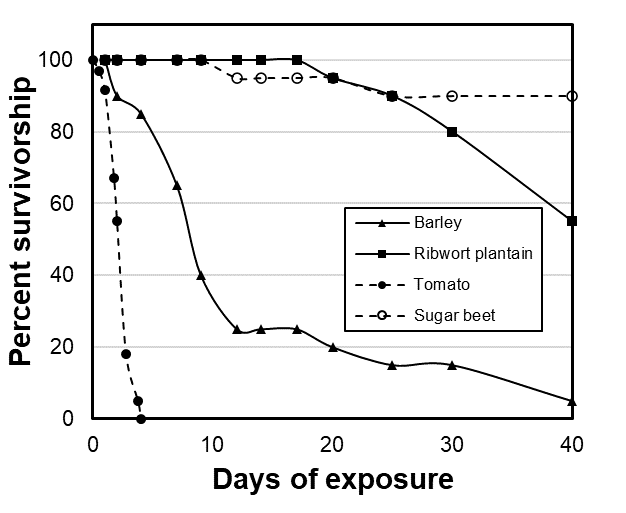


**Figure S2**. Probing preference of non-viruliferous beet leafhoppers between tomato and trap crop candidates in dual-choice experiments. Data are presented as mean ± SE. Asterisks indicate significant differences (*p<0.05, **p<0.01, ***p<0.001, NS not significant).

**
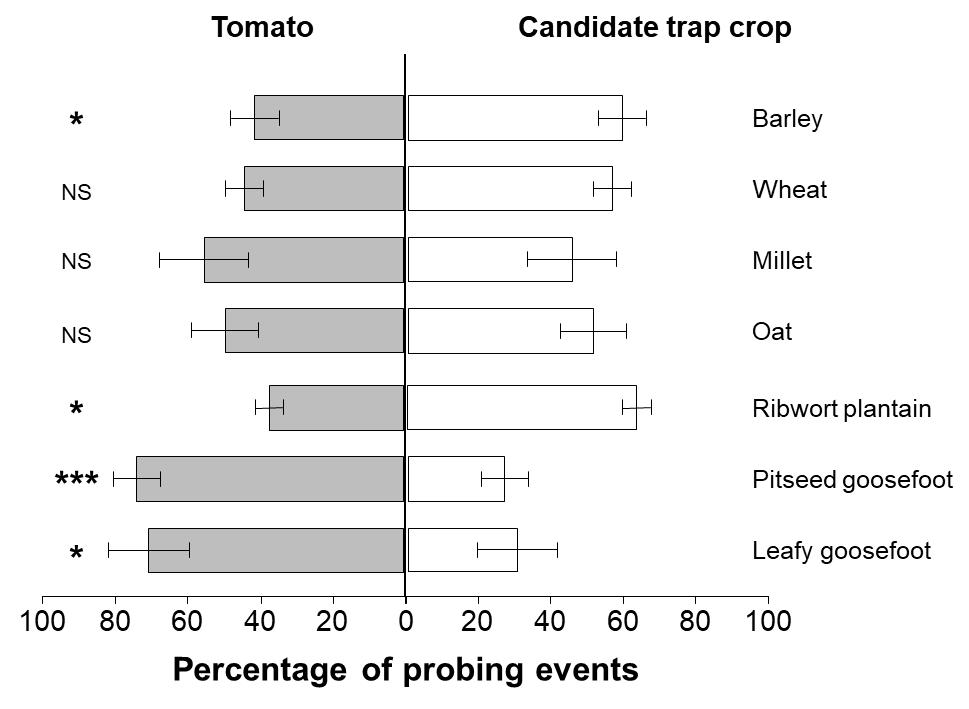
**

**Table S1.** Number of probing events and eggs (mean ± SE) on each plant species in the dual-choice experiments.

| Plant combination | Beet leafhopper | Plant | Number of probing events^1^ | Number of eggs^2^ |
| --- | --- | --- | --- | --- |
| Tomato | Viruliferous | Tomato | 103.1 ± 15.68ab | 1.2 ± 0.40ab |
| vs. |  | Ribwort plantain | 99.3 ± 9.64 | 10.72 ± 1.38 |
| Ribwort plantain | Nonviruliferous | Tomato | 74.9 ± 10.85a | 0.76 ± 0.32a |
|  |  | Ribwort plantain | 117.4 ± 10.96 | 11.76 ± 2.03 |
| Tomato | Viruliferous | Tomato | 144.4 ± 13.23b | 2.52 ± 0.55b |
| vs. |  | Barley | 123.3 ± 12.73 | 0.48 ± 0.13 |
| Barley | Nonviruliferous | Tomato | 107.5 ± 7.52ab | 1.52 ± 0.44ab |
|  |  | Barley | 165.9 ± 12.17 | 0.2 ± 0.10 |

^1,2^Within a column means for tomato with the same letter indicates no significant difference in ANOVA, Tukey test (*P* < 0.05). There was no significant difference in the number of probing events and eggs for other plants (P > 0.05).

**References**

1 Munyaneza, J. E. & Upton, J. E. Beet leafhopper (Hemiptera: Cicadellidae) settling behavior, survival, and reproduction on selected host plants. J. Econ. Entomol. 98, 1824-1830, doi:10.1093/jee/98.6.1824 (2005).
